# Impact of Essential and Toxic Trace Elements on Cervical Premalignant Lesions

**DOI:** 10.3390/biomedicines13123015

**Published:** 2025-12-09

**Authors:** Jovana Kocić, Nebojša Zečević, Jovana Jagodić, Dejan Mihajlović, Marko Dzuverović, Nenad Pavlović, Jelena Kotur-Stevuljević, Dragan Manojlović, Aleksandar Stojsavljević

**Affiliations:** 1 Clinic for Gynecology and Obstetrics “Narodni Front”, Kraljice Natalije 62, 11000 Belgrade, Serbia; gasicjovana@gmail.com (J.K.); nebojsa.zecevic1@gmail.com (N.Z.); dzuverovic.marko@gmail.com (M.D.); nenadpavlovic8822@gmail.com (N.P.); 2 Faculty of Medicine, University of Belgrade, Doktora Subotića 8, 11000 Belgrade, Serbia; 3 Faculty of Chemistry, University of Belgrade, Studentski Trg 12-16, 11000 Belgrade, Serbia; manojlo@chem.bg.ac.rs; 4 Clinical Hospital Center Kosovska Mitrovica, Anri Dinana 10, 38220 Kosovska Mitrovica, Serbia; dejan.mihajlovic@med.pr.ac.rs; 5 Faculty of Pharmacy, University of Belgrade, Vojvode Stepe 450, 11000 Belgrade, Serbia; jkotur@pharmacy.bg.ac.rs; 6 Innovative Centre, Faculty of Chemistry, University of Belgrade, Studentski Trg 12-16, 11000 Belgrade, Serbia; aleksandars@chem.bg.ac.rs

**Keywords:** cervical intraepithelial neoplasia (CIN), trace elements, essentiality/toxicity, CIN tissues

## Abstract

**Bacground/Objectives:** Cervical intraepithelial neoplasia (CIN) comprises a range of precancerous cervical lesions, and timely detection and intervention are essential to avert the development to invasive cervical cancer. Our previous study showed specific elemental alterations in the serum of patients with diagnosed CIN. In this study, we aimed to determine the levels of trace elements (Be, Cr, Mn, Co, Ni, Cu, Zn, Se, Cd, Tl, Pb, Rb, Sr, Mo, Th, and U) in more valuable materials, cervical tissue samples collected from patients diagnosed with CIN 2 and 3 (n = 60). **Methods:** The control group consisted of healthy, pathologically unaltered samples from the same patients (n = 60). The concentrations of all trace elements were determined using inductively coupled plasma–mass spectrometry (ICP-MS). Key demographic and clinical data were statistically analyzed in the context of trace element levels in cervical tissues. **Results:** We discovered that CIN 2 and CIN 3 tissues had significantly higher concentrations of essential trace elements Cr, Co, Se, and Mo, and toxic trace elements Be, Ni, and Cd compared to controls. The findings of this study highlight the differences in trace element concentrations in CIN tissue samples compared to controls. **Conclusions:** The presented results suggest the possible role of trace elements in the pathophysiological processes that lead to neoplasms in cervical tissues. The results provide initial and pivotal insight into the trace element concentrations in CIN tissues, which could aid further studies regarding cervical neoplasms and their pathogenesis.

## 1. Introduction

Cervical intraepithelial neoplasia (CIN) or cervical dysplasia is a term that describes the altered cells on the surface of the cervix [1]. It is a premalignant condition that is usually caused by infection with specific types of human papillomavirus (HPV) [2]. Cervical intraepithelial neoplasia is typically asymptomatic and, if untreated, can progress to cervical cancer (CC) based on microscopic findings graded from 1 to 3 [3]. CIN 1 has mildly changed cells (altered cells affecting approximately one-third of the thickness of the epithelium) and is less likely to become cancerous than CIN 2 (altered cells affecting approximately one-third to two-thirds of the epithelium) or CIN 3 (altered cells affecting more than two-thirds of the epithelium) [4,5]. In fact, CIN 1 usually resolves spontaneously. However, CIN 2 and CIN 3 are more likely to require treatment to prevent CC [6].

Human papillomavirus is the most common sexually transmitted infection worldwide. There are over 100 strains of HPV [7]. Some strains, particularly HPV-16 and HPV-18, are more likely to infect the reproductive tract and cause CIN. HPV prevention includes HPV vaccination, safe intercourse, and regular Pap smears [8]. Therefore, CIN affects sexually active people. Each year, approximately 250,000 to 1 million women are diagnosed with CIN [9]. CIN most often occurs in women of reproductive age, particularly between the ages of 25 and 35 [10]. A diagnosis of CIN is made if a Pap smear is inconclusive or reveals abnormal cells and further examination of the cervix by colposcopy [11]. Additionally, a biopsy can be helpful, as can deoxyribonucleic acid (DNA) testing. Treatment for CIN 2 and CIN 3 involves several options, the most common of which is conization (removal of a cone of tissue containing abnormal cells and further testing). Removing or destroying abnormal cells reduces the risk of CC by 95% in women with high-grade dysplasia in the first eight years after treatment [6,12].

In addition to HPV, multiple environmental factors are also shown to be involved in the risk of cervical cancer development [13]. The complex, multi-stage process of carcinogenesis is commonly acknowledged to be influenced by many environmental variables, including exposure to trace elements [14].

Essential trace elements (such as Mn, Cr, Co, Zn, Se, Cu, and Mo) have many roles in metabolic processes, serving as important components of enzyme reactions, and contributing to the functionality of the overall body, including reproductive health [15]. They have roles in antioxidative defense, cell proliferation and differentiation, regulation of hormones, and many others [16]. Differences in essential element levels have been strongly linked to the development and progression of multiple diseases [15,17]. On the other hand, exposure to toxic trace elements (Pb, Be, Cd, U) has been associated with oxidative stress, endocrine system disruption, and DNA damage, and some of them have been proven to contribute to the onset of various diseases, including cancer [16,17].

The concentrations and functions of trace elements in CIN are not yet fully understood. Since tissues can accumulate elements over time, analyzing cervical tissue samples provides more detailed and reliable information about elemental status than clinical samples such as serum, plasma, or urine. Therefore, this study aimed to compare the levels of essential and toxic trace elements (beryllium (Be), chromium (Cr), manganese (Mn), cobalt (Co), nickel (Ni), copper (Cu), zinc (Zn), selenium (Se), cadmium (Cd), thallium (Tl), lead (Pb), rubidium (Rb), strontium (Sr), molybdenum (Mo), thorium (Th), and uranium (U)) in CIN 2, CIN 2/3, and CIN 3-affected cervical tissues with those in adjacent, unaffected (self-control) tissues. By using actual cervical tissue, the most clinically meaningful material, this study can more accurately clarify the role of trace elements in CIN and support future biochemical, clinical, and other research.

## 2. Materials and Methods

### 2.1. Sample Collection

The present study included 60 patients with diagnosed CIN 2, CIN 2/3, and CIN 3. The diagnosis was initially made through Pap smear and colposcopic examination, and subsequently confirmed by biopsy following radio-knife conization. Conization, performed under general anesthesia, was used to excise cervical tissue. Each conization specimen included the transformation zone and surrounding cervical tissue. Control tissue samples were collected from the same patients, serving as a self-control group. All tissue samples were submitted to pathologists to confirm the initial diagnosis. Pathologists evaluated the tissue, separating healthy (control) tissue from pathologically altered tissue. Pathologists evaluated all conization specimens according to standard histopathological procedures. After sampling, tissues were placed for 20 h in formalin, and serial sections were fixed and placed in molds. The molds were left in formalin, and then they were placed in a fixation device and poured with paraffin, in order to obtain tissue sections of 3 mm. After fixation in formalin and routine paraffin embedding, the tissue was sectioned and stained with hematoxylin and eosin (H&E). Under light microscopy, pathologists examined the entire specimen to identify areas of normal cervical epithelium and areas exhibiting histopathological features of CIN 2, CIN 2/3 or CIN 3. The differentiation between healthy (control) tissue and pathologically altered tissue was based on established morphological criteria, including the degree of epithelial atypia, loss of maturation, mitotic activity, and architectural disturbances. The separation of healthy versus affected tissue was therefore performed microscopically during histological examination, without any additional cutting beyond the standard diagnostic processing of conization samples. This approach reflects routine diagnostic practice in gynecologic pathology.

All samples for trace elements assessment were stored in sterile tubes at −80 °C until analysis.

A questionnaire was used to collect key patient information, including age, smoking status, and CIN grade (CIN 2, CIN 2/3 or CIN 3). Patients who reported smoking confirmed consuming at least 10 cigarettes per day for the previous 10 years. To reduce potential confounding factors affecting trace element levels, strict exclusion criteria were applied. Patients were excluded if they had diabetes, cardiovascular disease, any neoplastic disease, or hepatic and/or renal failure. Individuals working in the chemical industry or in other environments with possible occupational exposure to trace elements (through inhalation or ingestion) were also excluded. The study received ethical approval from the Ethics Committee of the Clinic for Gynecology and Obstetrics at Narodni Front (number 24/461, from 1 June 2023) in Belgrade, Serbia. Prior to participation, all patients were fully informed about the purpose and procedures of the study. Participation was entirely voluntary, and written informed consent was obtained from each patient. Confidentiality and anonymity were strictly maintained, and all data were handled in accordance with applicable privacy and data-protection standards. The study was conducted in compliance with institutional guidelines and the ethical principles outlined in the Declaration of Helsinki.

### 2.2. Analysis of Trace Elements

CIN tissue samples and control tissue samples were first weighed accurately on an analytical balance (Thermo Fisher Scientific, Warrington, UK) and placed in a microwave cuvette. High-grade, ultra-pure reagents were added to the cuvettes (4:1, v:v, concentrated nitric acid: concentrated hydrogen peroxide), and the samples were decomposed with microwave digestion. Microwave digestion was carried out using the following temperature program: heating to 85 °C for 4 min, then to 130 °C for 4 min, followed by 5 min at 180 °C, and finally held at 180 °C for an additional 20 min. Once the samples were fully digested and cooled, they were transferred into volumetric flasks and diluted to a final volume of 25 mL using ultrapure water (resistance of 18.2 MΩ).

Selected trace elements (Be, Cr, Mn, Co, Ni, Cu, Zn, Se, Cd, Tl, Pb, Rb, Sr, Mo, Th, and U) (Table S1) were quantified by applying inductively coupled plasma mass spectrometry (ICP-MS) (iCAP Qc, Thermo Fisher Scientific, Warrington, UK). All elements analyzed using ICP-MS were measured under optimized conditions based on the kinetic energy discrimination (KED) technique. To minimize ion signal variability due to matrix effects, an internal standard (IS) solution—containing 50 µg/L of ^7^Li, and ^45^Sc, and 10 µg/L of ^71^Ga, ^89^Y, ^115^In, ^159^Tb, and ^208^Bi—was uniformly introduced into all samples, including blanks, calibration standards, and clinical samples, through a separate channel of the peristaltic pump.

External calibration was performed using two independent standard series prepared from stock solutions: the EPA Method Standard for low-level elemental calibration (10 mg/L, VHG Labs, Manchester, NH, USA) and the Comprehensive Mix A Standard (10 mg/L, VHG Labs, Manchester, NH, USA). Instrumental parameters for the ICP-MS analysis are provided in Table S1. To evaluate analytical accuracy, a certified reference material (CRM) bovine liver (NIST 1577c, Gaithersburg, MD, USA; Table S2) was analyzed. The CRM was prepared strictly according to the manufacturer’s instructions and processed in the same manner as the tissue samples. For elements that could not be reliably quantified using the CRM, the standard-addition method was applied (see Table S3).

### 2.3. Statistical Analysis

Shapiro-Wilks and Kolmogorov–Smirnov tests were used for the distribution testing, according to the number of subjects in both subgroups (control tissues and CIN tissues). Mann–Whitney U test and chi-squared test were employed to compare parameters between groups for the case of continuous and categorical variables, respectively. The principal component analysis with varimax-normalized rotation was used to extract the factors, which reduced the number of variables by grouping them into a smaller number of entities with comparable variability. The main conditions for PCA are Kaiser–Meyer–Olkin’s measure of sampling adequacy larger than 0.500 and Bartlett’s test of sphericity’s *p* value below 0.050. We used the predefined number of factors (n = 3) as the modus operandi for the factors’ selection, while the variables with factor loadings below 0.5 were excluded from further analysis. PASW^®^ Statistic v.18 (Chicago, IL, USA) software was used for data analysis; *p* values < 0.05 for all analyses were considered statistically significant.

## 3. Results

Table 1 presents the median values of quantified trace elements, along with their P25–P95 ranges and the corresponding *p*-values from the Mann–Whitney U test.

Zn was shown to be the most prevalent element in both control and CIN tissue samples, while Tl was the least prevalent. Control tissue samples exhibited the following element distribution: Zn> Se > Sr > Mn > Cr > Ni > Cu > Pb > Mo > Rb > Co > Cd > Th > U > Be > Tl. The CIN tissue samples showed a slightly different distribution: Zn > Cd > Sr > Se > Ni > Mn > Pb > Cu > Mo > Co > Rb > Th > Cd > U > Be > Tl. Statistically significant differences were observed in several trace elements: the concentrations of essential Cr, Co, Se, and Mo were significantly lower in control tissue samples, while toxic trace elements Be, Ni, and Cd were significantly higher in CIN tissue samples.

**Figure 1:**
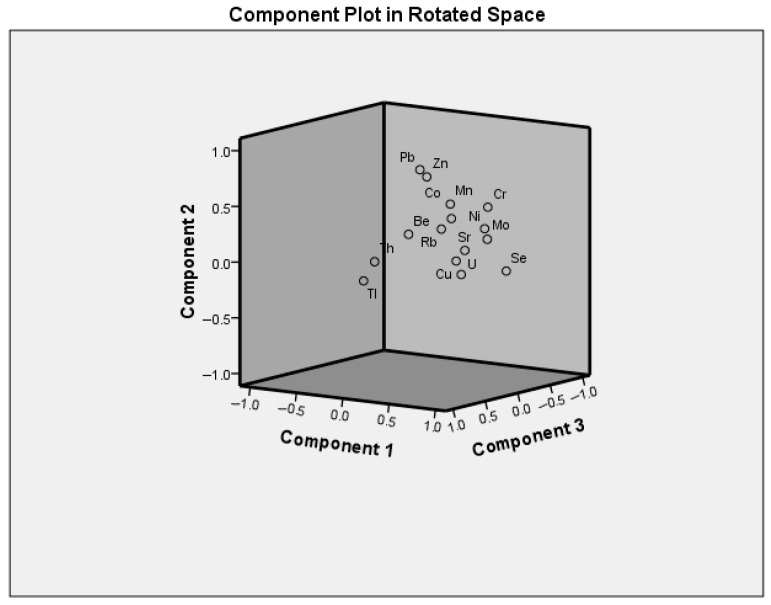
Component plot in rotation space.

The factorial analysis for the analyzed tissue samples is presented in Table 2. Factorial analysis was adequate (KMO = 0.773) and Bartlett’s test of sphericity (*p* < 0.001). A total variance explained was 67% with distinct factors ranging from 35%, 16% and 16%, respectively, for Factors 1–3 (Figure 1). Factor 1 included 10 out of the 15 trace elements examined, most of which are essential elements (Se, Cr, Mo, Mn, Cu, and Co). Furthermore, high loadings were observed for the toxic elements Ni and U, along with Se, Cr, Mo, and Sr. The clustering of such a wide range of elements suggests that Factor 1 likely reflects a broad influence on the metabolism caused by the presence of the disease. In contrast, Factor 2 consisted of only essential Zn and toxic Pb, an interesting combination that could indicate a disruption of element homeostasis that is influenced by CIN. Factor 3 involved Tl, Th, and Be, toxic elements that could possibly indicate increased oxidative stress due to the presence of CIN.

## 4. Discussion

### 4.1. Essential Trace Elements

Chromium has an essential role in the regulation of cholesterol levels and blood sugar [18]. However, occupational exposure to Cr has been linked to decreased fertility in women [19]. Our results showed that Cr was around 4-fold higher in CIN tissue samples compared to controls. Information about Cr levels in CIN tissues is scarce in the literature. Nonetheless, some studies have examined different human disorders, including several types of cancer, that indicated the potential for Cr to accumulate in diverse tissues and perhaps contribute to cancer initiation [20,21,22]. Future studies on Cr levels in CIN tissues could provide important insights regarding its role in cervical neoplasia.

As an essential trace element, Mn plays various roles in the body, including regulation of glucose and lipid metabolisms, immune response, development of the skeletal system, activity of enzymes, and many others [23]. Although our results showed around 2-fold higher Mn in cases than controls, there was no significant statistical difference between these two groups. Nonetheless, higher Mn could indicate increased oxidative stress in CIN tissues.

Cobalt is a significant component of cobalamin (vitamin B12), which plays a critical role in the metabolism of folic and fatty acids [24]. Our results showed that CIN tissues exhibited 3-fold higher Co concentrations compared to controls. Research published by Hunek et al. [25] mentioned that the increase in the expression of glucose transporter 1 (GLUT1) can be positively associated with hypoxia caused by Co. They reported that healthy cervical tissue exhibited the lowest expression of this protein, while CIN showed higher levels. Furthermore, Hunek et al. mentioned that Co-induced hypoxia influences cell proliferation and angiogenesis that are associated with cancer cells [25]. Further studies are encouraged on a larger scale of samples to examine the exact role of Co in the onset and progression of CIN.

Among its numerous roles, Cu influences the regulation of blood pressure and heart rate, production of erythrocytes, development of bones and connective tissues, and immune response [26]. Dysregulation in Cu concentrations has been observed in various human diseases, including cervical, ovarian, and endometrial cancers, as well as in endometriosis [27]. Our findings revealed similar concentrations across CIN tissues and controls, suggesting that this element does not significantly affect CIN.

As an essential trace element, Zn plays a crucial role in transcription factors and numerous enzymes. Furthermore, among its many roles, Zn regulation includes DNA replication, oxidative stress response, repair of DNA damage, cell cycle regulation, and cell apoptosis [28]. Although CIN tissues had around 2-fold higher Zn concentrations compared to controls, there was no observed statistical significance. However, our previous work indicated significantly higher Zn concentrations in serum samples of CIN patients [29].

Selenium has a role in DNA damage prevention, redox regulation and it exhibits chemopreventive effects [28]. Furthermore, Se plays a crucial role in preventing the peroxidation of polyunsaturated fatty acids in the form of a glutathione peroxidase cofactor, resulting in a reduced risk of cell membrane damage [30]. Piekutowski et al. reported increased Se concentrations in cervical and uterine tumor tissues as well as in benign uterine tumors compared to controls [31]. Our results showed significantly higher Se concentrations in CIN tissues than in controls. Higher concentrations of Se in pathologically altered tissues could indicate a compensatory enhancement of antioxidative defenses, most likely stemming from the increased oxidative stress found in pathologically altered tissues [32]. Furthermore, our previous work indicated significantly lower Se concentrations in sera samples of CIN patients [29]. By comparing our current findings with our prior ones, it can be hypothesized that CIN tissues extract Se from the bloodstream for their progression.

Molybdenum was 3-fold higher in CIN tissues than in controls, indicating its importance in the CIN pathogenesis. Our previous paper that investigated serum levels of trace elements in CIN patients showed that Mo was significantly decreased in CIN patients compared to controls [29]. Since Mo plays an important part in various enzymes and oxidative defense [33], CIN’s increased oxidative stress could cause Mo to be drawn from the bloodstream and into the CIN tissues. The exact mechanisms and role of Mo in this disease are still unknown, and further studies are encouraged to figure out its role in CIN.

### 4.2. Toxic Trace Elements

Recent research has pinpointed that Ni has a detrimental effect on the epigenome. This toxic trace element could cause hypermethylation of DNA, histone modifications and could also interfere with microRNA activity [34]. These changes, influenced by Ni, could contribute to the development and progression of tumors [35]. In our previous research, we observed significantly lower Ni concentrations in sera samples of CIN patients [29]. However, our current results showed that Ni was around 4-fold higher in CIN samples compared to controls, indicating its importance in CIN pathophysiology. In summary, our findings suggest that CIN tissue could have an increased tendency to accumulate Ni from the bloodstream. However, the underlying mechanisms and the potential biological role of Ni in the development or progression of CIN remain unclear. Given that Ni exposure causes the generation of ROS and, in turn, activates inflammatory signaling pathways [36], it can be assumed that Ni can contribute to tumorigenesis in the female reproductive tract. Supporting this notion, Canaz et al. reported higher Ni concentrations in both epithelial ovarian cancer and borderline ovarian tumor compared to controls [36]. As Ni has now been implicated in several female reproductive system malignancies, its exact role warrants further and more detailed investigation.

Cadmium and its compounds have been classified as group 1 of human carcinogens by the International Agency for Research on Cancer (IARC). Furthermore, studies have identified Cd as an endocrine disruptor, since it can imitate the effects of estrogen in the body [37]. Our results showed that Cd was significantly higher in the CIN tissue group compared to controls. Higher Cd was also reported in neoplastic endometrial tissues by Rzymski et al. [38]. Although the exact role of Cd in CIN remains uncertain, our findings point to its potential significance in this condition, emphasizing the need for additional research to determine Cd’s contribution to cervical neoplastic transformations.

Considering the links between Be, lung cancer, and chronic beryllium disease, Be has been classified as a Group 1 human carcinogen by IARC [39]. However, its role in the onset and progression of CIN remains largely unexplored. Our results indicated approximately 2-fold higher Be concentrations in CIN tissue samples compared to controls. While Be carcinogenicity is well documented in several circumstances, especially occupational exposure, its function in cervical neoplastic transformation remains unknown. Given the probable methods by which Be may influence carcinogenesis, such as DNA damage [40], more research is needed to investigate its possible role in CIN.

The International Agency for Research on Cancer has classified Pb in the group 2B, as possibly carcinogenic to humans [41]. Literary data have shown a link between Pb and gastrointestinal, lung, bladder, and head-neck cancers [42]. Furthermore, occupational and environmental exposure to Pb can disrupt the production and regulation of sex hormones and menstrual cycles. Lead exposure has also been linked to reduced fertility and impaired pregnancy outcomes [37]. Although our results showed higher Pb concentrations in CIN tissues, the difference was not statistically significant. However, our previous findings showed significantly higher Pb concentrations in sera samples of CIN patients compared to controls [29], indicating potential systemic accumulation. Zhang et al. suggested that there is a link between the presence of CIN and Pb accumulation [42]. Taken together, the presented data suggest that Pb may have a role in CIN pathogenesis, necessitating additional larger-scale research into its impact and clinical implications.

### 4.3. PCA Analysis

To the best of our knowledge, this is the first study that evaluated concentrations of trace elements in CIN tissue samples. Therefore, principal component analysis (PCA) analysis conducted in previous investigations on CIN examined different parameters. Devi et al. conducted a PCA analysis of CIN tissues, invasive cancer tissues, and controls, focusing on NADH, flavins, porphyrin, and collagen [43]. On the other hand, Sahoo et al. applied PCA analysis to show the difference between epithelial hyperplasia tissue samples and CIN-1 cervical tissue samples [44]. Our results indicated that factor 1 in the PCA analysis explained 35% of the variance, with the majority of the investigated elements being grouped. These results could indicate the influence of the existing disease on the body and could potentially reflect oxidative stress as an underlying contributing factor. Factor 2 explained 16% of the variance, with toxic Pb and essential Zn grouped in this cluster, while Factor 3 grouped Th, Tl, and Be (16% of variance). Further studies are encouraged on this topic to further elucidate the roles of the investigated trace elements in CIN and possibly single out biomarkers in this disease.

### 4.4. Limitations

This study provides valuable insight into the status of trace elements in CIN tissues and could support future research into cervical pathologies. However, the study is limited by its sample size of 60 CIN tissue specimens. Additional investigations with larger cohorts are planned to more comprehensively evaluate the role of trace elements in CIN pathology.

Furthermore, the Pap smear and colposcopic examination have several well-recognized limitations, including the potential for false-negative results, limited sensitivity for detecting certain cervical abnormalities, and the inherently subjective nature of colposcopic interpretation, which may contribute to diagnostic variability.

## 5. Conclusions

The presented study showcases the first insight into the concentrations of selected trace elements in CIN tissue samples and healthy controls. The analysis of toxic and essential trace elements (Be, Cr, Mn, Co, Ni, Cu, Zn, Se, Cd, Tl, Pb, Rb, Sr, Mo, Th, and U) was performed by the utilization of ICP-MS. Our findings revealed that control tissue samples had significantly lower concentrations of Cr, Co, Se, Mo, Be, Ni, and Cd than CIN tissue samples. These findings suggest that disruptions in trace element homeostasis, together with HPV infection, could play a role in the development or progression of cervical neoplasms. The observed elemental alterations could reflect metabolic changes associated with neoplastic transformation or contribute to the cellular processes involved in the onset of dysplasia. Considering the findings from the literature on trace elements in ovarian, uterine, and endometrial cancers, the potential role of trace elements in cervical neoplasias warrants further investigation. This work provides valuable insight into the potential involvement of trace elements in cervical pathology. The presented findings lay the groundwork for future investigations into the role of trace elements in CIN pathogenesis, particularly future studies involving larger sample groups.

## Figures and Tables

**Table 1:** Median values together with ranges (ng/g) for tissue samples of the control group and CIN patients. Significant values of the Mann–Whitney U test are given in **bold**.

Parameter (Trace Elements)	Control Group (Unit ng/g)	Patients (Unit ng/g)	Mann–Whitney U Test (*p* Value)
Be	0.017 (0.012–0.027)	0.024 (0.014–0.041)	**0.048**
Cr	7.0 (4.2–12.9)	31.2 (4.7–49.2)	**0.003**
Mn	9.3 (4.6–14.9)	15.5 (6.5–24.3)	0.080
Co	0.272 (0.178–0.482)	0.653 (0.370–1.018)	**<0.001**
Ni	6.66 (3.58–16.49)	22.08 (9.33–31.45)	**0.004**
Cu	6.27 (4.09–10.70)	6.41 (4.19–8.35)	0.428
Zn	180.7 (118.4–309.3)	278.6 (141.1–545.7)	0.141
Se	21.80 (14.67–31.38)	25.74 (15.48–41.20)	**0.033**
Cd	0.061 (0.032–0.097)	0.072 (0.033–0.102)	**0.034**
Tl	0.012 (0.007–0.021)	0.011 (0.008–0.015)	0.948
Pb	5.05 (2.94–11.94)	7.10 (3.52–9.93)	0.078
Rb	0.345 (0.174–0.543)	0.394 (0.285–0.621)	0.230
Sr	16.76 (10.46–45.01)	29.60 (15.99–46.59)	0.654
Mo	0.865 (0.704–1.077)	2.664 (1.119–4.663)	**0.004**
Th	0.055 (0.031–0.141)	0.080 (0.056–0.150)	0.545
U	0.046 (0.034–0.072)	0.066 (0.045–0.086)	0.135

**Table 2:** Factorial analysis for trace elements measured in cervical tissue samples of CIN patients.

Factors	Parameters (Trace Elements) Extracted in Factors	Loadings	Variability (%) Total: 67
1.	Ni	0.825	35
	Se	0.795	
	U	0.789	
	Cr	0.782	
	Mo	0.778	
	Sr	0.753	
	Mn	0.616	
	Cu	0.599	
	Co	0.592	
	Rb	0.584	
2.	Pb	0.788	16
	Zn	0.735	
3.	Tl	0.791	16
	Th	0.736	
	Be	0.631	

## Data Availability

All data is available from the corresponding per request.

## Supplementary Materials

The following supporting information can be downloaded at https://www.mdpi.com/article/10.3390/biomedicines13123015/s1, Table S1: ICP-MS parameters; Table S2: Recovery values (R) for investigated elements obtained from CRM (NIST 1577c bovine liver) analysis by ICP-MS; Table S3: The standard addition recovery (R) experiment for selected trace elements in CIN tissues.
