# Supplementary material for: Impact of Essential and Toxic Trace Elements on Cervical Premalignant Lesions
Source: Biomedicines. 2025 Dec 9;13(12):3015. doi: 10.3390/biomedicines13123015 (PMC12730413; doi:10.3390/biomedicines13123015)
Supplement: Supplementary file 1 [file biomedicines-13-03015-s001.zip › biomedicines-3930250-supplementary.pdf]

Supplementary material

Impact of essential and toxic trace elements on cervical premalignant lesions

J. Kocić[1], N. Zečević[1,2], J. Jagodić[3*], D. Mihajlović[4], M. Dzuverović[1], N. Pavlović[1], J. Kotur-Stevuljević[5], D. Manojlović[3], A. Stojsavljević[6]

1 Clinic for Gynecology and Obstetrics "Narodni front", Kraljice Natalije 62, Belgrade, Serbia
2 University of Belgrade, Faculty of Medicine, Doktora Subotića 8, Belgrade, Serbia
3 University of Belgrade, Faculty of Chemistry, Studentski trg 12-16, Belgrade, Serbia
4 Clinical Hospital Center Kosovska Mitrovica, Anri Dinana 10, Kosovska Mitrovica, Serbia
5 University of Belgrade, Faculty of Pharmacy, Vojvode Stepe 450, Belgrade, Serbia
6 Innovative Centre of the Faculty of Chemistry, University of Belgrade, Studentski trg 12-16, Belgrade, Serbia
Corresponding author: *E-mail: jovanaj@chem.bg.ac.rs

Table S1. ICP-MS parameters.

Parameter	ICP-MS
RF power (W)	1550
Argon gas flow rates (L/min)	
Coolant	0.80
Auxiliary	1.13
Transport	16.0
Nebulizer	PFA-ST MicroFlow
Spray chamber	Glass cyclonic
Replicates	3
Analyzed analyte	Isotopes (amu)
	^{9}Be ^{53}Cr ^{55}Mn
	^{59}Co ^{60}Ni ^{65}Cu
	^{68}Zn $^{78/82}$Se ^{85}Rb
	^{86}Sr ^{92}Mo ^{203}Tl
	^{208}Pb ^{232}Th ^{238}U

Table S2. Recovery values (R) for investigated elements obtained from CRM (NIST 1577c bovine liver) analysis by ICP-MS. .

Element	Certified value	Found value	R (%)
Co (mg/kg)	0.300	0.354	118
Cu (mg/kg)	275.2	271.8	98.8
Mn (mg/kg)	10.46	9.56	91.4
Mo (mg/kg)	3.30	2.79	84.5
Se (mg/kg)	2.031	1.808	89.0
Zn (mg/kg)	181.1	186.3	103
Cd (μg/kg)	97.0	94.9	97.8
Cr (μg/kg)	53	55.1	104
Pb (μg/kg)	62.8	59.7	95.0
Ni (μg/kg)	44.5	46.2	104
Sr (μg/kg)	95.3	93.7	98.3

Table S3. The standard addition recovery (R) experiment for selected trace elements in CIN tissues.

Element	Internal standard	R(%)	
Be	In	96.7*	95.8**
Tl	In	98.3*	99.8**
Rb	In	98.6*	101**
Th	In	99.4*	101**
U	In	101*	98.9**

*spiked with 10 μg/L; ** spiked with 20 μg/L.
